# Behavioral Inhibition Places Preschoolers at Risk for Reduced Social Competence, but Only in the Context of Other Temperamental Traits

**DOI:** 10.3390/children13010042

**Published:** 2025-12-28

**Authors:** Hailey Fleece, Hedwig Teglasi

**Affiliations:** Department of Counseling, Higher Education, and Special Education, University of Maryland, College Park, MD 20742, USA; hfleece@umd.edu

**Keywords:** behavioral inhibition, temperament profiles, latent profile analysis, social competence, early childhood development, emotion regulation, reactivity, preschoolers, social-emotional functioning, shyness

## Abstract

**Background/Objectives**: Behavioral inhibition (BI) has been extensively studied as an early-appearing risk factor for adverse developmental outcomes. One pathway through which BI may confer risk is via reduced competence to interact effectively with peers. Research demonstrating concurrent relations between BI and social competence supports this pathway, yet not all inhibited children experience social difficulties. This study adopted a person-centered approach to examine heterogeneity of temperament traits within a highly inhibited preschool sample and to identify how broader temperament traits contribute to variability in social functioning. **Methods**: Parents of preschoolers (*N* = 254) who met criteria for BI (≥85th percentile on the Behavioral Inhibition Questionnaire) completed measures of their child’s temperament (Children’s Behavior Questionnaire) and social competence (Social Skills Improvement System). Latent Profile Analysis was conducted using six temperament traits reflecting regulation and reactivity (anger, attentional focusing, inhibitory control, high-intensity pleasure, perceptual sensitivity, and approach). Profile differences in social competence were examined using multivariate analyses controlling for age and gender. **Results**: A three-profile solution emerged: Regulated, Unregulated and Angry, and Typical BI. Profile membership accounted for almost 37% of the variance in social skills scores. The Regulated group, marked by high attentional and inhibitory control and low anger, demonstrated the strongest social skills and lowest internalizing and externalizing problems. The Unregulated and Angry group, characterized by high anger and poor regulation, exhibited the greatest social difficulties. BI level itself did not significantly differentiate profiles or predict social competence. **Conclusions**: Findings underscore that BI is not a uniform risk factor but joins with other temperamental traits to shape social outcomes. Level of BI did not differentiate profiles or relate to social functioning, highlighting the importance of considering co-occurring regulatory and reactive traits to explain variability in outcomes among inhibited children. Identifying specific temperamental constellations may enhance early identification and inform targeted interventions for socially at-risk inhibited children.

## 1. Introduction

Behavioral inhibition (BI) is a biologically based temperament trait characterized by heightened sensitivity to novelty and fearfulness in social contexts [1]. Approximately 15–20% of young children display BI, and these early tendencies are moderately stable across development [2]. BI is widely regarded as an early risk factor for later psychopathology, particularly anxiety disorders, in part because it often co-occurs with social withdrawal, peer rejection, and difficulties forming friendships [3,4]. One developmental pathway through which BI may confer risk is through reduced social competence, defined as the use of social and emotional skills to achieve personal goals effectively across contexts [5]. Social competence in preschool is a robust predictor of both academic success and mental health outcomes [6]. Yet despite decades of research linking BI with social difficulties, important gaps remain in understanding variability within BI populations. BI is typically treated as a single risk factor for concurrent social difficulties and longer-term adverse outcomes, an approach that conceals heterogeneity among children with BI. The current study aims to identify dispositional factors accompanying BI that exacerbate or mitigate its associated social risks.

Social functioning has been established as a critical contributor to children’s overall well-being. Skills such as communication, cooperation with peers, empathy, and self-control are crucial for success in both academic and peer-group settings [6]. Beginning in preschool, inhibited children often withdraw from the peer group, which reduces opportunities to practice socially effective behaviors and can lead to long-lasting deficits in social competence [4]. This sequence accords with the Cascade Model of Development [7], which posits that early appearing vulnerabilities in one domain influence day to day transactions in ways that may detract from functioning in other domains, thereby amplifying its contribution to adverse outcomes over time.

The Developmental Cascade Model of Behavioral Inhibition describes the pathways and consequences of BI in early childhood [8,9,10]. According to this model, early manifestations of BI influence how children interact with their environment, peers, and caregivers, which sets off a cascade of effects across various domains of development. Children who are anxiously withdrawn report higher levels of peer rejection, greater feelings of loneliness, and fewer friendships [4]. Observational studies show that behaviorally inhibited children are more likely to have their social overtures ignored or dismissed by peers and are less frequently approached by others compared to their less inhibited peers [10,11]. In line with cascade models of development, concurrent social functioning reflects learning from past interactions and shapes children’s subsequent social experiences.

Much of the existing literature treats BI as a unitary construct, relying on variable-centered methods that examine its associations with outcomes across entire samples. This approach obscures meaningful heterogeneity, as not all behaviorally inhibited children experience social or emotional problems, and many maintain positive peer relationships [12]. This variability reflects the principle of multifinality, where early appearing traits can lead to diverse outcomes depending on co-occurring characteristics and contexts [13]. A growing body of research suggests that other temperamental traits, such as attentional control, effortful regulation, and anger, interact with BI to influence developmental pathways [14]. For example, regulation can buffer risk, whereas anger may exacerbate it by disrupting interactions with peers and caregivers. However, anger has rarely been studied in the context of BI, despite its well-established links with both externalizing and internalizing symptoms [15].

A broader literature on temperament also highlights common protective and risk factors for social competence. Protective factors include traits that support flexible responses to social demands, such as positive emotionality and attentional shifting, whereas high negative emotionality and low effortful control consistently predict poorer competence [16]. These findings come largely from variable-centered research in both community and BI samples, but they highlight the traits that may combine in different ways to shape inhibited children’s developmental outcomes. Importantly, this work suggests that interventions should ultimately be tailored to individual temperamental constellations rather than single traits.

A person-centered approach offers a way to capture this heterogeneity among children with BI based on constellations of temperamental traits rather than a single characteristic [17]. Latent Profile Analysis (LPA) is particularly suited to this task because it can uncover naturally occurring configurations of temperament that might explain why some inhibited children thrive socially while others struggle. Person-centered approaches align with goodness-of-fit models, which emphasize developmental outcomes as the product of the interplay between relevant dispositional traits and environments [18]. In each context, a temperamental tendency such as BI joins with other dispositional traits to influence functioning. Examining profiles of temperament within behaviorally inhibited children provides a way to capture this interplay among dispositional traits and to clarify when inhibition is a risk factor and when it is not.

This study aims to identify profiles of temperamental dispositions among children with BI and to investigate how membership in profile groups relates to children’s concurrent social functioning and thereby to increase understanding of the variability in outcomes among inhibited children. Examining individual differences in the temperamental characteristics of inhibited children is useful to learn more about why some children with BI experience social and emotional difficulties whereas others do not. To accomplish these aims, we address two broad research questions and hypotheses:

1. What profiles emerge when examining the temperament traits of behaviorally inhibited preschool-aged children?

We expected to identify distinct subgroups that differ in regulatory capacity, emotional reactivity, and approach/avoidance tendencies.

2. Does social competence vary across these profiles?

We hypothesized that social competence would vary meaningfully across profiles, with profiles characterized by anger showing lower social competence, and profiles with higher inhibitory control showing greater social competence.

## 2. Materials and Methods

### 2.1. Participants

The parents and guardians of inhibited children were recruited via email, listserv, and in-person flyer requesting the participation of parents of young children. Participants were required to meet the following inclusion criteria: be the parent or guardian of a child who is between the ages of 3- and 6-years old (36 to 83 months of age); report concerns about their child exhibiting characteristics of BI; rate their child at the 85th percentile or higher on the Behavioral Inhibition Questionnaire; and live in the United States. Exclusion criteria included a diagnosis of autism, developmental delay, or intellectual disability.

Three outliers were removed from the dataset due to large Mahalanobis distances. The remaining sample consisted of 254 parents. Most of the participants were the biological or adoptive mother of the behaviorally inhibited child (93.37%) and the rest were the biological fathers (5.07%), legal guardians (1.17%), or stepmothers (0.39%) of the behaviorally inhibited child. Additionally, most of the sample was White (76.66%). The sample also included Asian (11.72%), Hispanic or Latinx (5.86%), African American or Black (5.47%), and Native American (0.39%) parents. Parents in the sample were highly educated, with 49.02% having a graduate degree, 34.20% having a bachelor’s degree, and 4.28% having an associate’s degree. Fewer than 15% of parents had not received a college diploma (7.00% Some College, 5.44% High School). Families had a median household income of $100,000 to $200,000 per year. Children’s ages ranged from 36 to 83 months (M = 53.61, SD = 12.73). Slightly over half of children were girls (55.7%). On average, children spent about 3 years (M = 2.98, SD = 1.67) in a childcare setting that included peers, with 97% of children having spent at least one year in daycare, preschool, or elementary school. Children each had an average of one sibling.

### 2.2. Measures

*Child Behavioral Inhibition*: The Behavioral Inhibition Questionnaire (BIQ) was administered to measure levels of inhibition in the study sample [19]. The BIQ is a 30-item questionnaire that measures the frequency of behaviors associated with BI on a 7-point Likert scale from 1 = Hardly Ever to 7 = Almost Always. The BIQ comprises two factors, Novel Social Inhibition which measures BI in response to Adults, Peers, and Performance demands and Novel Situational Inhibition, which measures BI in response to New Situations, Physical, and Parental Separation/Preschool. Descriptive statistics of each scale in the present sample are summarized in Table 1. All subscales of the BIQ demonstrated skew and kurtosis values that did not exceed ±1, indicating approximately normal distributions. In the current sample, the BIQ also demonstrated good internal consistency ranging from Ω = 0.74 to Ω = 0.85. No data were missing from the BIQ.

Child Temperament: The Children’s Behavior Questionnaire-Short Form (CBQ-SF) is a 94-item broadband measure of temperament in 3- to 7-year-old children [20]. Caregivers were asked to rate their child’s behavior within the past six months on a seven-point scale ranging from 1 = Extremely Untrue of Your Child to 7 = Extremely True of Your Child. The CBQ comprises 15 subscales that load onto three dimensions of temperament: Surgency/ Extraversion, Negative Affectivity, and Effortful Control. Descriptive statistics of each scale in the present sample can be found in Table 2. All subscales of the CBQ demonstrated skew and kurtosis values that did not exceed ±1, indicating approximately normal distributions. Most variables demonstrated internal consistency ranging from Ω = 0.61 to Ω = 0.82. Two scales, Sadness and Low Intensity Pleasure, fell below Ω = 0.60 and were excluded from analyses. From the full dataset, a total of 86 item responses were missing, and in alignment with CBQ scoring guidelines, mean substitution was used, in which each missing item was replaced with the mean of the other items within the same subscale for that participant.

Child Social Functioning: The Social Skills Improvement System-Rating Scale (SSIS-RS) is a 79-item broadband measure of Social Skills (Communication, Cooperation, Assertion, Responsibility, Empathy, Engagement, and Self-control) and Problem Behaviors (Externalizing, Internalizing) in children [21]. Parents rated the frequency of their child’s behavior in the past two months on 4-point Likert scales from 1 = Never to 4 = Always. Internal consistency, skew, and kurtosis were calculated in the current sample. See Table 3 for a summary of statistics. All subscales on the SSIS demonstrated acceptable normality (Skew and Kurtosis values between −1.00 and 1.00) and good internal consistencies, ranging from Ω = 0.70 to Ω = 0.88. From the full dataset, there were 8 individual items missing. In line with scoring guidelines provided by the SSIS authors, these missing responses were substituted with a value of two for missing items on the social skills scale and a value of one for the problem behaviors scale [21].

### 2.3. Procedures for Statistical Analysis

To address the first research question, a Latent Profile Analysis (LPA) was conducted. LPA is a person-centered statistical technique that clusters homogeneous subgroups of individuals within a larger population [22]. Preliminary analyses initially explored inclusion of all 15 CBQ subscales in the LPA to capture broad temperament patterns among behaviorally inhibited children. However, models including all CBQ subscales yielded unstable profile solutions with limited interpretability, likely because the number of indicators created overly complex models for the available sample size.

To improve stability and interpretability, the number of CBQ variables included in the LPA was reduced. Selection of indicators was guided by theory, prior literature, and characteristics of the current sample. Attentional Focusing, Inhibitory Control, and Perceptual Sensitivity were retained to capture regulatory and sensitivity-related processes; Anger/Frustration was included as a reactive trait known to exacerbate BI-related risk; and High Intensity Pleasure and Approach/Positive Anticipation were selected to reflect engagement with stimulation and novelty [14,15]. Additional criteria included adequate variability and reliability within the current sample: subscales with restricted range (e.g., Shyness, Impulsivity) or inadequate internal consistency (Sadness, Low Intensity Pleasure) were excluded. Lastly, the CBQ comprises three overarching factors: Negative Affectivity, Extraversion/Surgency, and Effortful Control. Given that the initial aim was to explore all 15 variables, it was important to have representation from all three primary factors. Taking all inclusion criteria into consideration, the final variables chosen for the LPA included Anger, Approach, Attentional Focusing, Inhibitory Control, Perceptual Sensitivity, and High Intensity Pleasure. The LPA was conducted in Mplus Version 8 [23] using the six chosen subscales of the Children’s Behavior Questionnaire to find groups of inhibited children who have different temperamental profiles. All models were estimated using 1000 starts and 250 final stage optimizations.

An exploratory approach was used where multiple models were fit to the data to determine the optimal number of profiles [24]. In each LPA model, means of the indicators were allowed to vary freely across the three latent classes, whereas variances and covariances were constrained to be equal across classes. This procedure was selected to promote model convergence and interpretability and is consistent with recommendations for modest samples where analyses are focused on how groups differ on average. Models with one to six profiles were compared and the most theoretically defensible model with the fewest number of profiles that best fit the data was selected. Multinomial logistic regression tested whether child age, sex, and BI-level predicted profile membership.

To address the second research question, profile differences in social-emotional functioning and problem behaviors were examined using BCH-adjusted distal outcome models. This approach accounts for classification uncertainty inherent in latent profile solutions and avoids bias associated with assigning individuals to their most likely class. Distal outcomes included SSIS Social Skills subscales (Communication, Cooperation, Assertion, Responsibility, Empathy, Engagement, and Self-Control) as well as Externalizing and Internalizing Behavior scores. BCH analyses were conducted using Latent GOLD, using posterior class membership probabilities. Child age and sex were included as covariates. Wald chi-square tests were used to evaluate omnibus differences across profiles, followed by BCH-adjusted pairwise comparisons to examine specific between-profile differences.

## 3. Results

### 3.1. Preliminary Analyses

Participants qualified to participate in the study by scoring at or above the 85th percentile on any of the following: overall BIQ score, social novelty, or situational novelty scores. In the final sample, the average overall BIQ score fell at the 97th percentile (M = 153.96, SD = 18.69). On the social novelty scale, the average participant fell at the 93rd percentile (M = 77.46, SD = 10.59). On the situational novelty scale, the average participant scored at the 94th percentile (M = 76.50, SD = 11.81). BI has historically been associated with impaired social competence [12]. Therefore, a simple linear regression was conducted to examine the relation between the BIQ overall score and SSIS Total Social Skills score. The regression model was not statistically significant (F(1, 252) = 3.28, *p* = 0.071), suggesting that in the current, highly restricted sample, overall BI did not predict social skills.

### 3.2. Results of Research Question #1: What Profiles Emerge When Examining the Temperament Traits of Behaviorally Inhibited Preschool Aged Children?

#### 3.2.1. The Class Enumeration Process

LPA analyses with one to six profiles were estimated using the six CBQ variables. Each solution was examined by considering the Sample-Adjusted Bayesian Information Criterion (SABIC) statistic, Adjusted Lo-Mendell Rubin (LMR) likelihood ratio test statistic, class sizes, and theoretical plausibility of group profiles. A summary of model fit indices and class proportions for the 1–6 class solutions is presented in Table 4.

After carefully considering each solution, the three-profile solution was determined to have the best fit. First, SABIC values decreased as the number of classes included in the model increased, providing evidence for models with a larger number of classes. This information was considered alongside the Lo-Mendell-Rubin (LMR) test, which assesses whether there is a statistically significant improvement in model fit between k − 1 classes and k classes. None of the LMR tests reached statistical significance at the 0.05 level. However, the LMR test at the 3-class level, yielded a *p*-value of 0.09, slightly above the 0.05 threshold. This suggests that while not statistically significant, there is some indication that the 3-class model may be preferable over the 2-class model.

The number of cases in each class was also considered. Based on the rule of thumb suggested by Lubke and Neale [25], solutions with profiles containing fewer than 25 cases were examined for their theoretical relevance. The simpler models, including the 2-class, 3-class, and 4-class solutions, provided more balanced and meaningful class distributions compared to the class counts for the 5 and 6-class solutions.

Given ambiguous fit indices, theoretical plausibility was a key factor in evaluating the 2-, 3-, and 4-class solutions. When comparing the theoretical plausibility between the 2- and 3-class solutions, the 2-class solution was found to group together distinct categories of children. For example, children with moderate and high levels of attention and inhibitory control were placed in the same group, which was inconsistent with theoretical expectations. In contrast, the 4-class solution included classes that were not theoretically distinct from one another, suggesting a lack of meaningful differentiation. The 3-class solution, however, was found to have distinct groupings that were theoretically defensible, aligning with previous research on temperament. Taken together, this evidence suggested that the three-profile solution provided the best balance between statistical fit and theoretical coherence, making it the most appropriate choice for further analysis.

#### 3.2.2. Descriptive Statistics: The Three Class Solution

The three-profile solution is shown in Table 5. Each participant was assigned to the profile in which they had the largest probability of being a member. The three classes were treated as groups and labeled based on their most apparent temperamental characteristics.

Class 1—Regulated. The first profile comprised 50 children (19.69%) who were labeled as “Regulated.” This group of children was marked by lower levels of Anger and High Intensity Pleasure and higher Attentional Focusing and Inhibitory Control, as measured as being at least 0.5 standard deviations away from the mean of the overall sample. There were 19 boys and 31 girls in this group. The average child age was 4 years and 10 months old.

Class 2—Unregulated and Angry. The second profile contained 55 children (21.65%) and was labeled as “Unregulated and Angry.” In comparison to the overall sample, these children had higher levels of Anger and lower levels of Attentional Focusing and Inhibitory Control. Although not significantly different from the overall sample, the Unregulated and Angry children had higher levels of High Intensity Pleasure compared to the Regulated children. There were 33 boys and 20 girls in this group. The average child age was 4 years and 1 month old.

Class 3—Typical BI. The third profile contained 149 children (58.66%). This group was labeled as “Typical BI” because it contained the largest percentage of children and did not vary significantly from the overall sample in any of the six temperament variables. There were 59 boys and 87 girls in this group and the average child age was 4 years and 5 months old.

#### 3.2.3. LPA Covariate Effects

Given evidence that age, gender, and BI influence children’s social–emotional functioning [26,27], multinomial logistic regression tested these covariates as predictors of profile membership. BI was dichotomized at the sample mean (Very High vs. Moderately High). Relatively higher or lower levels of BI did not significantly differentiate the three groups. Compared with the Unregulated and Angry group, the Regulated group included older children (OR = 1.43; 95% CI [1.02, 2.08]). Older age also predicted Regulated over Typical BI membership (OR = 1.45; 95% CI [1.02, 2.08]). Relative to the Unregulated and Angry group, the Typical BI group comprised older children (OR = 1.61; 95% CI [1.06, 2.50]) and fewer boys (OR = 0.34; 95% CI [0.15, 0.74]).

### 3.3. Research Question #2: How Are the BI Profiles Related to Concurrent Social and Emotional Functioning?

Latent profile membership (Regulated, Unregulated and Angry, and Typical BI) was examined as a predictor of children’s social skills and problem behaviors using BCH-adjusted distal outcome models. This approach accounts for potential bias associated with classification uncertainty while preserving the latent profile solution. Posterior class membership probabilities indicated that children were assigned to profiles with a high degree of certainty (average posterior probabilities ranged from 0.91 to 0.93; see Table 6). Nevertheless, all distal outcome analyses were conducted using BCH-weighted means, rather than modal class assignment, to ensure unbiased estimation.

A BCH-adjusted model indicated a significant overall effect of latent profile membership on total social skills, controlling for child age and gender (Wald χ^2^(2) = 88.95, *p* < 0.001). Profile membership accounted for 36.84% of the variance in total social skills. Children in the Unregulated and Angry profile demonstrated the lowest total social skills, whereas children in the Regulated profile demonstrated the highest scores.

BCH-adjusted omnibus Wald tests revealed significant differences among profiles across all SSIS social skills (Communication, Cooperation, Assertion, Responsibility, Empathy, Self-Control, and Engagement) and problem behavior domains (Externalizing and Internalizing Behaviors), after adjusting for age and gender (see Table 7). Pairwise BCH comparisons indicated that, relative to both the Unregulated and Angry and Typical BI groups, children in the Regulated profile demonstrated significantly higher skills across all social skills and problem behavior domains. In comparison to the Typical BI group, children in the Unregulated and Angry group showed significantly lower Communication, Cooperation, Responsibility, Empathy, and Self Control skills, and higher levels of Externalizing behaviors. No significant differences were found between the Unregulated and Angry and Typical BI profiles on Assertion, Engagement, or Internalizing Behaviors.

#### Comparison of SSIS Across BI and Normative Sample

To further explore the relation between BI and social skills, an independent samples t-test examined whether children in the overall BI sample differed from the normative SSIS sample on social skills. Children with BI (M = 92.05, SD = 11.86, N = 254) scored significantly lower than the normative sample (M = 100, SD = 15, t = –6.03, *p* < 0.001). To explore subgroup differences, Welch’s t tests compared each BI subgroup with the normative sample (M = 100, SD = 15). The Regulated subgroup (M = 101.24, SD = 12.28) did not differ from the normative sample, t(104) = 0.58, *p* = 0.56. In contrast, the Unregulated and Angry subgroup (M = 84.40, SD = 9.52) scored significantly lower, t(77) = –7.58, d = −1.04, *p* < 0.001, and the Typical BI subgroup (M = 90.80, SD = 10.26) also scored significantly lower, t(251) = –6.07, d = −0.61, *p* < 0.001.

## 4. Discussion

This study advances our understanding of behavioral inhibition (BI) as an early appearing risk factor by showing that BI does not stand alone in relation to social emotional functioning but operates within broader temperament profiles. A large body of work has established BI as a temperament trait linked to negative social and emotional outcomes [3,14,28]. The Developmental Cascade Model [29], which posits that the impact of a single risk factor is magnified over time, provides a framework for understanding the pathway between early BI and subsequent social-emotional difficulties [8]. In accord with this framework, the impact of an early appearing vulnerability such as BI is compounded by affecting key areas of functioning, such as social competence, which is central to children’s well-being, as it supports peer acceptance, academic engagement, and long-term mental health [7,28]. Hence, current deficits in social competence may be viewed as outcomes of earlier developmental processes associated with BI that likely promote further negative cycles.

In the present sample of children with BI, overall social skills scores on the SSIS were significantly below the normative sample, highlighting the developmental vulnerabilities associated with this temperament trait. However, within this sample, level of BI did not correlate with social competence, a finding that supports the need to consider multiple influences, including the presence of distinct subtypes of BI that, along with other factors such as parenting and peer relationships, shape how early inhibition translates into maladaptive outcomes [8,30]. Understanding the various temperamental profiles within BI and their relation to concurrent social emotional functioning is crucial for developing more effective early interventions.

The current study posed two research questions. First, what temperament profiles emerge when examining behaviorally inhibited preschool-aged children? Second, are these temperament profiles related to concurrent social and emotional functioning?

### 4.1. Discussion of Research Question #1: What Profiles Emerge When Examining the Temperament Traits of Behaviorally Inhibited Preschool Aged Children?

The study used Latent Profile Analysis to identify subgroups of children based on selected temperamental characteristics that were hypothesized to shape the impact of BI on social functioning. The three-profile solution was identified as the best-fitting model. The Regulated group (20% of sample) was characterized by lower anger (full SD) and high intensity pleasure (half SD) and higher attentional focusing (half SD) and inhibitory control (full SD) than the sample mean. The Unregulated and Angry group (21% of sample) was characterized by higher anger (half SD), lower attentional focusing (full SD) and inhibitory control (half SD) compared to the sample mean, and elevated high intensity pleasure (full SD) compared to the Regulated group. The Typical BI group (59% of sample) was characterized by scores near the sample mean across these dimensions. Variations in levels of anger and frustration, attentional focus, inhibitory control, and high-intensity pleasure differentiated the subgroups. These traits represent meaningful dimensions of both reactivity and regulation that jointly shape a child’s behavior and personality [31].

Anger and frustration are highly reactive traits and, when paired with poor regulatory capacities as seen in the Unregulated and Angry subgroup, can lead to maladaptive outcomes such as social difficulties or externalizing behaviors, especially in children already predisposed to anxiety or withdrawal [32]. High intensity pleasure as a temperament trait may vary in adaptiveness depending on context. For some children, elevated high-intensity pleasure-seeking may support engagement and exploration, whereas for others, particularly when unregulated, it may amplify impulsivity or frustration during social challenges [33].

Attentional focusing and inhibitory control are core components of effortful control and were critical in differentiating the profiles in this study. High levels characterized the Regulated group and appeared to buffer the adverse effect of BI on social functioning. In the broader developmental literature, inhibitory control, along with other aspects of effortful control described by Rothbart and colleagues, is consistently linked to greater social competence and self-regulation [34,35,36]. More generally, effortful control is thought to support adaptive behavior by enabling children to regulate emotions, sustain attention, and flexibly modulate responses in social contexts [35]. However, the role of inhibitory control in behaviorally inhibited children is more complex and nuanced.

Several studies have found that among children high in BI, elevated inhibitory control may increase risk for anxiety symptoms [37,38]. However, these studies primarily focused on reactive control in laboratory settings, where inhibitory control is measured with Stroop-like tasks which require children to override an automatic response in favor of a subdominant one [38]. In contrast, the measure of inhibitory control used in this study captured its positive implications and reflected more planful, goal-directed control (e.g., Prepares for trips and outings by planning things s/he will need). According to the Detection and Dual Control framework [14], this distinction between reactive and planful inhibitory control is meaningful. Whereas planful control may promote adaptive functioning in the face of BI, reactive control may heighten risk. Thus, the emergence of inhibitory control as a key differentiator in the present profiles reflects its more adaptive, planful form, particularly when combined with other regulatory strengths such as attentional focusing.

Although Perceptual Sensitivity did not significantly differ between profiles, prior research suggests that it warrants consideration in the context of BI. Perceptual sensitivity reflects the tendency to detect subtle, low-intensity cues in the environment and may shape how children interpret novel or ambiguous social contexts. Heightened perceptual sensitivity may amplify attentional biases toward threat in children with BI [39], yet it may also serve as a protective factor when paired with strong regulatory capacities, given associations with empathy, conscientiousness, and prosocial awareness in broader samples [40,41]. In designing future research, it is important to consider challenges in the measurement of perceptual sensitivity, which may be less behaviorally observable and more context-dependent, with its impact changing based on environmental conditions and caregiver interpretations [39,42].

Approach/positive anticipation had the smallest variability of the six CBQ subscales chosen for the analyses, reducing its utility for subgroup differentiation. This range restriction is likely reflective of the highly inhibited population. The Approach/Positive Anticipation scale of the CBQ reflects outward excitement for positive anticipated events, with questions such as “Gets very enthusiastic about the things s/he does.” In novel and social situations, behaviorally inhibited children show lower positive affect and low approach behaviors with their surroundings [43].

An important source of evidence supporting the need for profiles was the finding that BIQ total scores did not emerge as a covariate in the profile analysis. The lack of association between level of BI and profile group membership suggests that once a child surpasses a high threshold of inhibition, which in this study was the 85th percentile, other temperament traits play a more influential role in shaping the child’s presentation. Prior research has examined single temperament traits, such as inhibitory control, within behaviorally inhibited populations [14,38]. The current study builds on these foundations by demonstrating the convergence of multiple temperament dimensions that meaningfully differentiates subgroups within a highly inhibited population.

Gender and age were associated with profile membership. Older children were more likely to belong to the Regulated group, a pattern consistent with the development of emotion regulation capacities during the preschool period [44]. Additionally, boys were more likely to be classified in the Unregulated and Angry group, aligning with previous research demonstrating that boys tend to have greater challenges with emotion regulation [45]. Importantly, even when controlling for gender and age, the three temperament profiles remained distinct, highlighting the robustness of these groupings and contributing to a growing body of work conceptualizing BI as a multifaceted construct [30].

### 4.2. Research Question #2: How Are the BI Profiles Related to Concurrent Social and Emotional Functioning?

Although this sample of children had significantly lower social skills scores on the SSIS than those in the normative national sample, the level of BI (total BIQ score) was not related to either the overall SSIS social skills scores or to the three identified profiles. This lack of association between level of BI and social functioning (despite considerable variability on both measures) and between BI and the profile groupings speaks to the need to consider other temperamental dispositions to understand the impact of BI on social functioning.

An important contribution of the current study is the finding that the impact of BI on social skills is not uniform across children and not necessarily associated with level of BI beyond the threshold for its identification. The effect of BI profile membership on social skills outcomes was substantial, with approximately 37% of the variance in SSIS scores explained by children’s profile classification, even when controlling for other influences like age and gender. Whereas BI is associated with reduced social skills on average, the degree of impairment depends on presence of other temperament traits, so that some groups of behaviorally inhibited children appeared to fare better socially than others.

The Regulated group, characterized by lower levels of anger and high-intensity pleasure and higher levels of attentional focusing and inhibitory control, demonstrated the highest levels of adaptive social skills. These children outperformed the other groups on multiple dimensions of social functioning, including communication, cooperation, assertion, responsibility, and empathy. Additionally, this group did not significantly differ from the normative SSIS sample on their overall social skills, suggesting that when BI is paired with regulatory strengths, such as attentional control, children may be better able to manage their shyness and fear and engage effectively in peer interactions.

The finding with respect to the Regulated profile aligns with literature emphasizing the protective role of self-regulation in the developmental outcomes of temperamentally vulnerable children [35]. However, other temperamental traits that are less studied such as relatively lower anger and higher enjoyment of activities that are highly stimulating may also be protective in the context of BI. Children with BI are frequently asked to participate in novel or demanding situations that they might otherwise prefer to avoid. Hence, enjoyment of high intensity stimulation, coupled with lower reactivity to frustration (anger), may therefore help them tolerate challenging social contexts and reduce risk for withdrawal.

The Unregulated and Angry group displayed significantly lower levels of adaptive social skills and elevated levels of both internalizing and externalizing difficulties relative to both the Regulated and the typical BI groups. These children were prone to frustration, negative reactivity, and low levels of regulation, which likely exacerbated their social challenges [35]. In comparison to the normative sample, the children in the Typical BI group had lower overall social skills, consistent with research and theory suggesting that many children with BI face social and emotional difficulties [10,11].

The differing profiles had a direct and measurable impact on how parents rated their children’s social competencies, indicating that their children’s underlying temperament traits shape their observable behavior in ways that are meaningful to caregivers. Substantial variation across profile groupings in social functioning speaks to the importance of adopting a nuanced and targeted approach to early intervention to avert negative trajectories. Behaviorally inhibited children who also demonstrate anger, poor attentional focusing, and low inhibitory control represent a particularly vulnerable subgroup in comparison to both typically developing children and their inhibited peers with better regulatory abilities. These children may benefit most from targeted early interventions aimed at improving emotion regulation and enhancing adaptive social skills.

### 4.3. Limitations and Future Research

Whereas this study provides valuable insights into the temperamental profiles of behaviorally inhibited children and their relations with social and emotional functioning, it is important to consider the constraints of the sample, which was limited by using a single rater, primarily including White, highly educated mothers. As a result, the findings may not be generalizable to more diverse populations, including those from different racial, ethnic, socioeconomic, or cultural backgrounds. The overrepresentation of mothers, particularly those with higher education levels, may also limit the ability to draw conclusions about child outcomes across different family structures.

The cross-sectional nature of the data limits the ability to draw conclusions about causality or to observe how the identified BI profiles evolve over time. Since the data were collected at a single point in time, the current study enabled conclusions about concurrent temperament and social functioning but did not allow for the assessment of whether certain temperamental profiles predict long-term social, emotional, or behavioral outcomes. Longitudinal studies would be necessary to confirm the stability of these profiles and to examine how they influence development in the long run.

For the CBQ, individual subscale-level mean substitution was used rather than the sample mean. This approach was selected because it best preserves the individual variability of participants’ scores, minimizes data loss, and aligns with CBQ scoring guidance. For the SSIS, missing responses were substituted using values based on the developers’ recommendations, which minimizes data loss and reflects the standardization sample’s average item scores. Although both methods were unlikely to have introduced significant bias due to the minimal level of missingness, they may still slightly attenuate variability or underestimate associations between variables. Future studies could consider using other approaches, such as full-information likelihood. In addition, some CBQ indicators included in the LPA demonstrated only moderate internal consistency (e.g., ω ≈ 0.60–0.70), which may have further attenuated distinctions between latent profiles and reduced class separation.

Finally, variances and covariances of the indicators in the LPA were constrained to be equal across classes. This approach was used to improve model convergence and interpretability and is consistent with recommendations for smaller samples where analyses are mainly focused on how groups differ on average. However, this approach assumes that all classes have the same variances and covariances, which could mask important differences in within-class variability. Future research using larger samples should consider relaxing these constraints to explore whether heterogeneity in variances or covariances improves model fit and interpretive precision. Replication with larger samples would also allow for more nuanced consideration of variables such as perceptual sensitivity, anger, and high intensity pleasure, which are currently not well studied in relation to BI.

## 5. Conclusions

Although this cross-sectional study cannot speak directly to developmental processes, the findings carry important implications for both developmental theory and applied practice. Participants in this study met criteria for BI and, as would be expected, were rated as lower in social competence than children in a normative sample. However, the emergence of distinct temperament profiles within a highly inhibited sample supports models that emphasize the heterogeneity of BI [8,30]. The profiles are characterized by unique combinations of regulatory and reactive traits that showed substantial relations with social functioning, which are among the strongest predictors of child well-being in the present and long term [46]. Although measured concurrently, the link between profile membership and social competence is presumably the outgrowth of prior developmental processes and influences subsequent processes.

For children with BI, understanding the influences of accompanying temperamental characteristics on concurrent social functioning would allow for more tailored interventions to improve transactions between the child and the context. Cascading effects of BI on development over time are thought to play a critical role in the adverse outcomes of children with BI [8]. By targeting proximal impacts of BI, such as negative peer transactions and deficits in social functioning, early interventions may head off more distal outcomes such as increased likelihood of social isolation and of internalizing symptoms such as anxiety and depression. However, the possibility of cascading influences of BI profiles warrants consideration of the extent to which short term improvements (in the fit) promote growth in other areas that are key to future functioning. In designing interventions, it may be helpful to consider how individuals’ moment to moment transactions in various contexts (goodness of fit models) translate into longer term developmental outcomes (cascade models).

The lack of association of BI level with social functioning and with profile grouping found in this sample, together with substantial associations of profile membership with social competence, suggests that children with similar BI tendencies differ in their developmental trajectories, depending on the presence of other dispositional traits. Person-centric approaches highlight the usefulness of developmental models of multifinality, goodness-of-fit, and cascading effects of risk factors to provide actionable insights for tailoring interventions.

## Figures and Tables

**Table 1:** The Behavioral Inhibition Questionnaire Descriptive Statistics.

BIQ Scale	*n*	Mean	SD	Skew	Kurtosis	Ω
Adults	254	5.74	1.06	−0.98	0.94	0.85
Peers	254	5.51	0.92	−0.29	−0.71	0.78
Performance	254	5.37	1.08	−0.19	−0.74	0.82
Separation/Preschool	254	5.23	1.24	−0.44	−0.14	0.84
New Situations	254	5.20	0.71	0.03	−0.31	0.74
Physical Challenges	254	3.50	1.28	0.24	−0.21	0.82
Total Score	254	5.13	0.62	0.32	−0.27	-

Note. Standard errors for skewness = 0.154 and kurtosis = 0.304.

**Table 2:** The Children’s Behavior Questionnaire-Short Form.

CBQ Scale	N	Mean	SD	Skew	Kurtosis	Ω
Activity Level	254	4.55	0.87	−0.33	0.33	0.64
Anger Frustration	254	4.55	1.24	−0.30	−0.31	0.82
Approach/Positive Anticipation	254	4.72	0.88	−0.22	0.05	0.61
Attentional Focusing	254	4.45	1.04	−0.14	−0.28	0.66
Discomfort	254	4.43	1.25	−0.37	−0.67	0.80
Falling Reactivity/Soothability	254	4.36	1.21	−0.33	−0.60	0.84
High Intensity Pleasure	254	4.56	1.11	−0.34	−0.11	0.71
Impulsivity	254	3.15	0.90	0.19	−0.59	0.68
Inhibitory Control	254	4.77	0.96	−0.27	−0.37	0.68
Low Intensity Pleasure	254	5.63	0.68	−0.51	0.10	0.57
Perceptual Sensitivity	254	5.28	0.88	−0.61	0.10	0.67
Sadness	254	4.46	0.84	−0.18	−0.30	0.53
Shyness	254	5.76	0.77	−0.64	0.33	0.69
Smiling/Laughter	254	5.39	1.05	−0.85	0.39	0.81
Fear	254	4.34	1.15	−0.29	−0.30	0.68

Note. Standard errors for skewness = 0.154 and kurtosis = 0.304.

**Table 3:** The Social Skills Improvement System Descriptive Statistics.

SSIS Scale	*n*	Mean	SD	Skew	Kurtosis	Ω
Communication	254	3.11	0.42	−0.26	0.62	0.70
Cooperation	254	3.07	0.53	−0.42	0.45	0.86
Assertion	254	2.91	0.46	−0.38	0.49	0.70
Responsibility	254	2.91	0.54	−0.44	0.60	0.85
Empathy	254	3.07	0.58	−0.35	0.08	0.88
Engagement	254	2.42	0.55	−0.08	−0.05	0.83
Self-Control	254	2.53	0.47	−0.16	0.34	0.77
Externalizing	254	1.81	0.46	0.69	0.85	0.88
Internalizing	254	1.77	0.43	0.66	0.21	0.77

Note. Standard errors for skewness = 0.154 and kurtosis = 0.304

**Table 4:** Summary of Temperament Solutions.

Model	LL	Npar	BIC	SABIC	Adj. LMR-LRT *p*-Value	Entropy	Class Counts
1-class	−2007.337	27	4164.181	4078.585	NA	NA	1: 254
2-class	−1996.701	34	4181.671	4073.884	0.2	0.704	1: 55 2: 199
3-class	−1982.713	41	4192.457	4062.478	0.09	0.801	1: 50 2: 55 3: 149
4-class	−1970.665	48	4207.121	4054.951	0.46	0.760	1: 34 2: 56 3: 50 4: 114
5-class	−1960.767	55	4226.087	4051.725	0.31	0.795	1: 4 2: 37 3: 53 4: 51 5: 109
6-class	−1951.063	62	4245.44	4048.887	0.28	0.809	1: 4 2: 30 3: 53 4: 52 5: 17 6: 98

**Table 5:** Final 3-Class Solution.

CBQ Scale	Mean	SD	1/2 SD Range	1 SD Range	LC1 M N = 50	LC2 M N = 55	LC3 M N = 149
Anger Frustration	4.55	1.24	(3.93–5.17)	(3.31–5.79)	2.99	5.72	4.69
Approach/Positive Anticipation	4.72	0.88	(4.28–5.16)	(3.84–5.6)	4.56	4.99	4.68
Attentional Focusing	4.45	1.04	(3.93–4.97)	(3.41–5.49)	5.06	3.38	4.64
Inhibitory Control	4.56	1.11	(4.00–5.12)	(3.45–5.67)	5.76	3.50	4.89
High Intensity Pleasure	4.77	0.96	(4.29–5.25)	(3.81–5.73)	4.05	5.23	4.49
Perceptual Sensitivity	5.28	0.88	(4.84–5.72)	(4.4–6.16)	5.58	5.36	5.14

**Table 6:** Posterior class membership probabilities.

Class	N	Mean Posterior Probability	SD
1: Regulated	50	0.93	0.10
2: Unregulated & Angry	55	0.92	0.13
3: Typical BI	149	0.91	0.11

**Table 7:** Means and Standard Errors of Social Skill, After Controlling for Age and Gender, by LPA class.

SSIS Scale	1: Regulated		2: Angry & Unregulated		3: Typical BI		Wald	*p*-Value
	Mean	SD	Mean	SD	Mean	SD		
Communication	17.14 _bc_	0.37	12.34 _ac_	0.43	14.93 _ab_	0.21	72.08	<0.001
Cooperation	15.5605 _bc_	0.34	8.58 _ac_	0.41	12.80 _ab_	0.21	171.33	<0.001
Assertion	14.4743 _bc_	0.44	12.34 _a_	0.55	13.40 _a_	0.27	9.78	0.008
Responsibility	14.3841 _bc_	0.38	8.11 _ac_	0.48	11.72 _ab_	0.22	105.45	<0.001
Empathy	14.81 _bc_	0.45	9.93 _ac_	0.50	12.53 _ab_	0.27	53.32	<0.001
Engagement	11.2602 _bc_	0.56	9.44 _a_	0.58	9.72 _a_	0.33	6.62	0.04
Self-Control	13.5374 _bc_	0.40	7.91 _ac_	0.49	10.73 _ab_	0.24	81.50	<0.001
**Total Social Skills**	**101.24 _bc_**	**2.19**	**84.40 _ac_**	**2.75**	**90.80 _ab_**	**0.93**	**88.94**	**<0.001**
Internalizing Bx	15.38 _bc_	0.53	19.06 _a_	0.559	18.12 _a_	0.41	25.61	<0.001
Externalizing Bx	16.25 _bc_	0.51	27.71 _ac_	0.8305	21.52 _ab_	0.40	153.06	<0.001

_a_ = significant difference from class 1 based on pairwise Wald tests (ps < 0.05). _b_ = significant difference from class 2 based on pairwise Wald tests (ps < 0.05). _c_ = significant difference from class 3 based on pairwise Wald tests (ps < 0.05).

## Data Availability

The datasets presented in this article are not readily available because the data are part of an ongoing study. Requests to access the datasets should be directed to hfleece@umd.edu.
